# Methods of Radiographic Measurements of Heart and Left Atrial Size in Dogs with and without Myxomatous Mitral Valve Disease: Intra- and Interobserver Agreement and Practicability of Different Methods

**DOI:** 10.3390/ani12192531

**Published:** 2022-09-22

**Authors:** Charanthorn Levicar, Ingo Nolte, José Luis Granados-Soler, Fritjof Freise, Jonathan Friedemann Raue, Jan-Peter Bach

**Affiliations:** 1 Clinic for Small Animals, University of Veterinary Medicine Hannover, Foundation, 30559 Hannover, Germany; 2 Institute for Biometry, Epidemiology and Information Processing, University of Veterinary Medicine Hannover, Foundation, 30559 Hannover, Germany

**Keywords:** vertebral heart size, radiographic left atrial dimension, left atrial width, vertebral left atrial size, observer agreement

## Abstract

**Simple Summary:**

In the present study, different radiographic measurements for evaluating the cardiac size and left atrial size in dogs with and without myxomatous mitral valve disease were evaluated. Veterinarians applied these measurements with different levels of experience to identify heart enlargement in conventional and inverted radiographs. The repeatability and ease of application were evaluated. It was shown that radiographic measurements can identify cardiac enlargement reliably and even potentially stage dogs with myxomatous mitral valve disease if echocardiography is unavailable. The measurements can be performed in conventional and inverted radiographs with reliable results by veterinarians with different degrees of experience.

**Abstract:**

Dogs suffering from Myxomatous Mitral Valve Disease (MMVD) show a potential heart enlargement, especially in the left atrium, detectable by radiography. Due to digital radiography, different radiographic measurements estimate cardiac size quite uncomplicatedly. The Vertebral Heart Size (VHS), Radiographic Left Atrial Dimension (RLAD), Left Atrial Width (LA_Width_), and the Vertebral Left Atrial Size (VLAS) used anatomical landmarks for measuring cardiac size in relation to the vertebral column. This study aimed to compare VHS, RLAD, LA_Width_, and VLAS measured in conventional and inverted radiographs by veterinarians with different levels of experience in healthy dogs and dogs with MMVD. The reliability and user-friendliness of these measurements were evaluated, and the staging was compared to the echocardiography staging. A total of 50 unaffected dogs and 150 dogs with MMVD in stages B1, B2, and C were assessed. Three veterinarians with different levels of experience examined 200 conventional radiographs and their corresponding inverted radiographs blinded to the echocardiographic and clinical examination results. Analyses were performed to compare the measurements’ grading and determine anatomical landmarks with measurement difficulties. Additionally, inter- and intraobserver agreement was assessed using intraclass correlation coefficient, and the agreement between radiographic and echocardiographic staging was compared using the kappa coefficient. The VHS, LA_Width_, and VLAS were easier to define than the RLAD. The interobserver agreement was almost perfect for VHS (0.962) and good for the other radiographic measurements (RLAD: 0.778, LA_Width_: 0.772, VLAS: 0.858). The VHS assigned the most dogs to the correct stage. However, VHS, RLAD, LA_Width_, and VLAS presented an almost perfect intraobserver agreement. The dorsal left atrial margin of the RLAD was the most difficult measurement point to identify. The VHS is the most reproducible radiographic method for measuring the canine heart size and shows the highest agreement with echocardiography. An observer-related influence could be detected for RLAD, LA_Width_, and VLAS.

## 1. Introduction

Assessing thoracic radiographs is of enormous diagnostic value in veterinary radiology and widespread use [1,2,3,4,5,6]. Apart from evaluating the lung field and the pulmonary vessels, information about the overall cardiac size and individual cardiac compartments can be obtained [4,5,7,8,9,10,11]. Especially the assessment of left atrial (LA) size is critical in many cardiac diseases like Myxomatous Mitral Valve Disease (MMVD), which is the most common cardiac disease in dogs [3,12]. To detect MMVD, the Consensus Statement of the American College of Veterinary Medicine (ACVIM) recommends the echocardiographic examination [3]. However, an echocardiographic examination is not always available. The importance of evaluating LA size is based on the fact that LA enlargement is a crucial factor in assessing the risk of present or future congestive heart failure (CHF) [3,7,13]. Additionally, detection of LA enlargement can help to distinguish stage B1 patients from stage B2 patients [14,15,16,17].

Due to these reasons, radiologic evidence of overall cardiomegaly and LA enlargement are criteria for initiating therapy in patients with asymptomatic MMVD [3,18]. Different radiographic signs (e.g., the elevation of the left mainstem bronchus or bulging of the cardiac silhouette in the LA area) have been described to detect LA enlargement [7,10,19]. However, evaluating these signs is highly subjective and can be imprecise [7,17]. To ensure an objective radiologic evaluation, cardiac size is commonly quantified with radiographic measurements using anatomical landmarks [20,21,22,23,24,25,26]. 

In the current consensus statement of the ACVIM, the use of the long-established Vertebral Heart Size (VHS) [20,27,28] and the more recent Vertebral Left Atrial Size (VLAS) [23] are proposed [3]. The VHS has been proving reliability in detecting general cardiomegaly [15,19,29], whereas the VLAS has been promising in determining the LA size and predicting a possible enlargement due to MMVD [23,30]. Apart from these radiographic measurements, the Radiographic Left Atrial Dimension (RLAD) and the Left Atrial Width (LA_Width_) demonstrated the potential to detect LA enlargement in preclinical MMVD patients [24,26]. Due to digital radiography, the cardiac size can be easily estimated using proposed radiographic measurements, and the contrast of the radiographs can be modified. Commonly in conventional radiographs, structures with high radiopacity like bones appear white, structures with low radiopacity like gas appear black, and soft tissues are presented in different shades of gray depending on their radiopacity [31]. Inversion of radiographs displays originally black structures as white and vice versa [32].

An important criterion for determining whether a radiographic measurement is adequate is investigating its agreement with actual LA size, which in a clinical setting is commonly measured via echocardiography [33]. This question has been examined in several previous studies [15,16,34,35]. Recently, the authors developed cut-off values measured by a specialized veterinarian and showed a lot of potential for staging MMVD patients [17].

In addition, reproducibility of measurements and easy applicability by observers with different levels of experience is crucial for determining the usefulness of a radiographic measurement in a clinical setting. The VHS has proven its reliability and reproducibility in several studies [15,19,29]. For RLAD and VLAS, a potential influence of the observers’ experience on the results of the measurements has been described [36,37]. Yet, comparable investigations for LA_Width_ in terms of reproducibility are still lacking. No previous study has methodically compared the reproducibility and applicability of the different methods for measuring cardiac and LA size.

To this end, the current study aimed to investigate the reproducibility of the mentioned radiographic measurements and compare them regarding echocardiographic staging and their ease of use, evaluated by three observers with different levels of experience. In addition, the individual radiographic landmarks were assessed regarding the ease of determining their exact position on radiographs. All measurements in conventional and inverted radiographs were compared to find differences in recognition of anatomical landmarks depending on the form of their presentation.

## 2. Materials and Methods

### 2.1. Animals

No institutional animal care and use approval were required for this retrospective study. During their visit, owner consent was obtained for all dogs. Digital thoracic radiographs in the right lateral recumbency of 200 dogs were obtained from the patient system of the Clinic for Small Animals, University of Veterinary Medicine Hannover, Foundation, Hannover, Germany. A total of 50 healthy dogs and 150 patients with different stages of MMVD were included. All dogs were subjected to a cardiac evaluation without sedation, including physical examination, radiography, and echocardiography with an electrocardiogram. Group allocation was based on clinical, radiographic, and echocardiographic findings (Echocardiographic system Vivid 7 or 9, GE Healthcare GmbH, Solingen, Germany), and patients were allocated to the different groups according to the latest consensus statement of the ACVIM [3]. A total of 50 dogs were included in each of the stages B1, B2, and C. Stage B1 had dogs with a systolic heart murmur and mitral valve regurgitation detected by Doppler echocardiography, but without echocardiographic and radiographic cardiomegaly. Stage B2 included asymptomatic patients with mitral valve regurgitation and significant enlargement of the LA and left ventricle in echocardiography described by a left atrium to aorta ratio (LA/Ao) ≥ 1.6 and normalized left ventricular internal dimension at the end diastole (LVIDDn: left ventricular internal dimension at end diastole (cm)/body weight (kg)^0.294^) ≥ 1.7 [38,39]. Dogs in stage C had mitral valve regurgitation with cardiomegaly and previous or current clinical signs of CHF. A reading of the echocardiographic measurements is displayed in Supplementary File S1. A total of 57 dogs allocated to stages B2 and C received medication, including diuretics, but no medication was changed between the examinations (Supplementary File S1). The control group included healthy dogs without clinical or echocardiographic signs of MMVD and normal thoracic radiographs.

### 2.2. Radiography

All images were acquired using a digital radiography system (Philips Bucky-Diagnost, Philips GmbH, Hamburg, Germany). Dogs were placed in right lateral recumbency with extended forelimbs. The radiographic settings were weight-specific according to previously defined weight tables of the radiologic department of the Clinic for Small Animals (Supplementary File S2).

For study inclusion, a maximum time interval of three days between radiographic and echocardiographic examination was allowed. Furthermore, only radiographs with the appropriate image quality of the patient for diagnostic evaluation positioning were included.

### 2.3. Observers

Three veterinarians with different levels of experience, including a recent graduate (CL, Observer 1), a veterinarian with more than 10 years of experience in small animal medicine with a focus on small animal cardiology (JPB, Observer 2), and a veterinary radiologist (German specialist in veterinary radiography, JFR, Observer 3) performed the measurements. All examiners were blinded regarding the group allocation of the patients on the radiographs.

### 2.4. Radiographic Measurements

The measurements were performed on a high-resolution monitor (Dell UltraSharp U2720Q with a 3840 × 2160 resolution at 60 fps, Dell Technologies Inc., Round Rock, TX, USA) using a DICOM viewer of a standard veterinary clinical software (easy image, VetZ GmbH, Isernhagen, Germany). The radiographs were inverted with the same clinical software. In conventional radiographs, structures with a high radiopacity, like bones, were shown in white, whereas structures with low radiopacity, like gas, were shown in black. The soft tissues were presented in different shades of gray. After inversion, the structures with high radiopacity appeared black and the structures with low radiopacity white. Measurements examples in the inverted radiographs are displayed in Supplementary File S3. The order of images was randomized using the rand function of standard computer software (Excel 2016, Microsoft Corporation, Redmond, WA, USA). The observers were blinded to the results of the clinical and echocardiographic examination. The following four radiographic measurements were obtained in each conventional and inverted radiograph. Measurements were applied as a Vertebral Scale, beginning at the cranial margin of the fourth thoracic vertebra, and recorded as vertebral units to one decimal place. One vertebral unit ran from the cranial margin of one vertebral body to the cranial body off the subsequent one [19].

**Figure 1:**
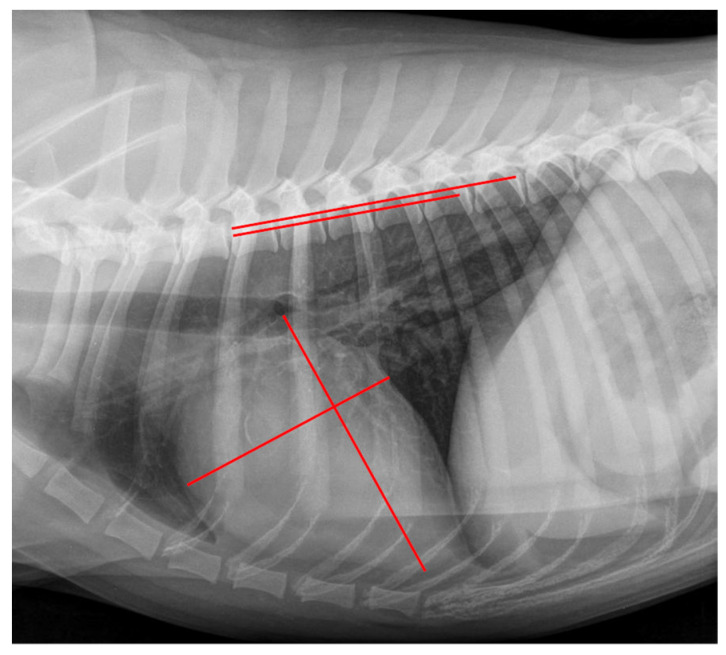
Measurement example of VHS. Right lateral thoracic radiograph displaying the Vertebral Heart Size (VHS) measurement in a dog of the control group. The long axis was drawn from the ventral margin of the carina tracheae to the most distal margin of the cardiac apex, and perpendicular to the short axis was drawn at the level of the ventral intersection of the caudal vena cava and the cardiac silhouette (red lines on cardiac silhouette). These lines were repositioned onto the vertebral column (red lines on vertebral column) beginning at the cranial margin of the fourth thoracic vertebrae. The VHS was 10.5 vertebral units.

The VHS was obtained as described by Buchanan et al. (1995) [20] and modified according to Jepsen-Grant et al. (2003) [28]. A measurement example can be seen in Figure 1. First, the long axis of the cardiac silhouette was measured from the ventral margin of the carina tracheae to the most distal margin of the cardiac apex. Second, the short axis was drawn at a 90° angle to the long axis and at the level of the ventral intersection of the caudal vena cava and the cardiac silhouette. Afterward, the length of both axes were transcribed onto the vertebral column as described above.

**Figure 2:**
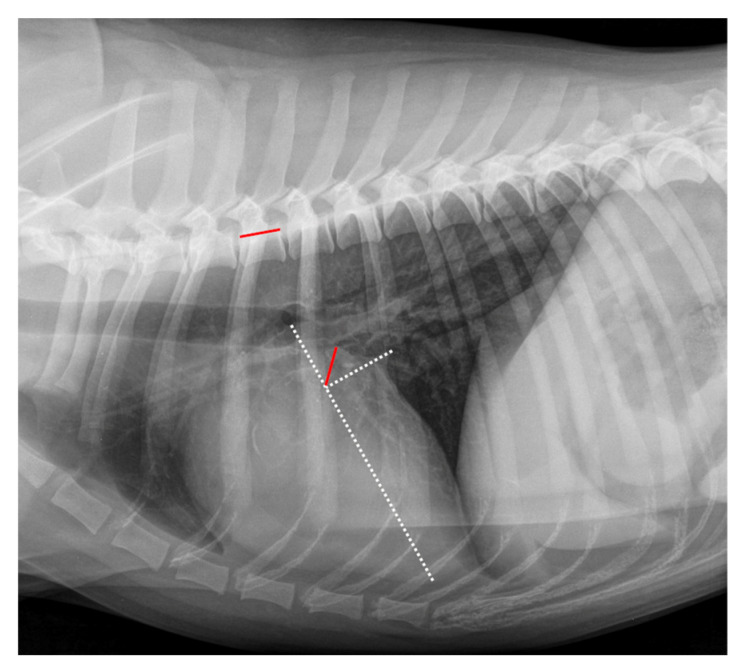
Radiographic Left Atrial Dimension (RLAD) measurement in the same right lateral thoracic radiograph seen in Figure 1. The long axis (white dotted line) was applied as described for the VHS measurement (Figure 1). The short axis (white dotted line) was drawn from dorsal intersection of the caudal vena cava and the cardiac silhouette to the long axis. The bisecting RLAD line was drawn from the intersection point to the dorsal margin of the left atrium (red line on cardiac silhouette). This line was transposed onto the vertebral column (red line on vertebral) as described in Figure 1. The RLAD was 0.8 vertebral units.

The RLAD was measured as described by Sanchez et al. (2018) [24]. An example of measurement is displayed in Figure 2. As a basis for measuring the RLAD, two axes had to be drawn similarly as described for the VHS. The long axis was drawn exactly as described for VHS. However, the short axis was drawn at the level of the dorsal intersection between the caudal vena cava and the cardiac silhouette and perpendicular to the long axis. Afterward, starting at the intersection point of the long and short axes, a bisecting line was drawn through the LA to its most dorsal margin. Finally, this line was transposed onto the cranial margin of the fourth thoracic vertebra, then the vertebral units were counted, and the result was recorded as RLAD.

**Figure 3:**
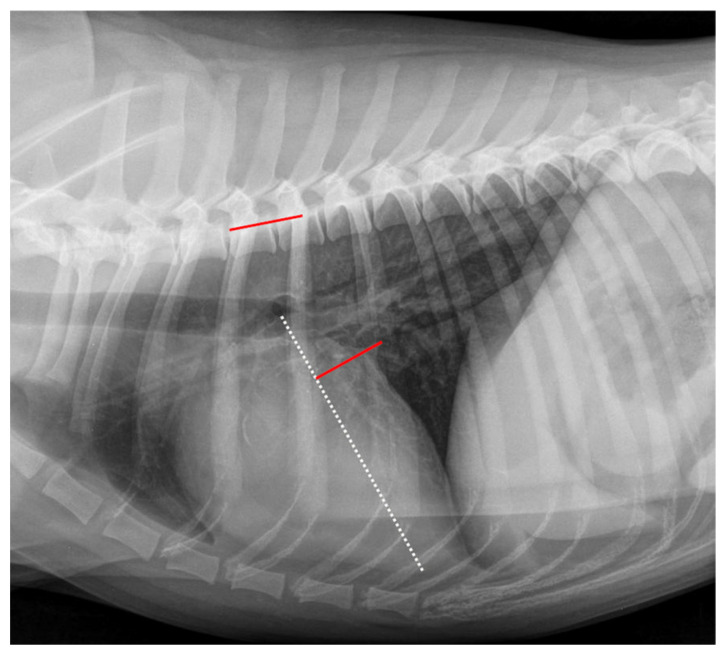
Measurement example of LA_Width._ The Left Atrial Width (LA_Width_) measurement in the same right lateral thoracic radiograph is shown in Figure 1. The long axis was measured as described in Figure 1 (white dotted line). The short axis was drawn at a 90° angle to the long axis at the height of the dorsal intersection between the cardiac silhouette and the caudal vena cava (red line on cardiac silhouette). This line was repositioned onto the vertebral column (red line on vertebral) as described in Figure 1. The LA_Width_ was 1.6 vertebral units.

The LA_Width_ was measured as described by Stepien et al. (2020) [26] and is shown in Figure 3. As a basis for measuring the RLAD, two axes had to be drawn similarly as described for the VHS. The long axis was measured for VHS and RLAD. Afterward, the LA_Width_ line was drawn at a 90° angle at the height of the dorsal intersection between the cardiac silhouette and the caudal vena cava. This line was finally applied to the vertebral column beginning at the fourth thoracic vertebra, and the number of encompassed vertebrae was recorded as LA_Width_.

**Figure 4:**
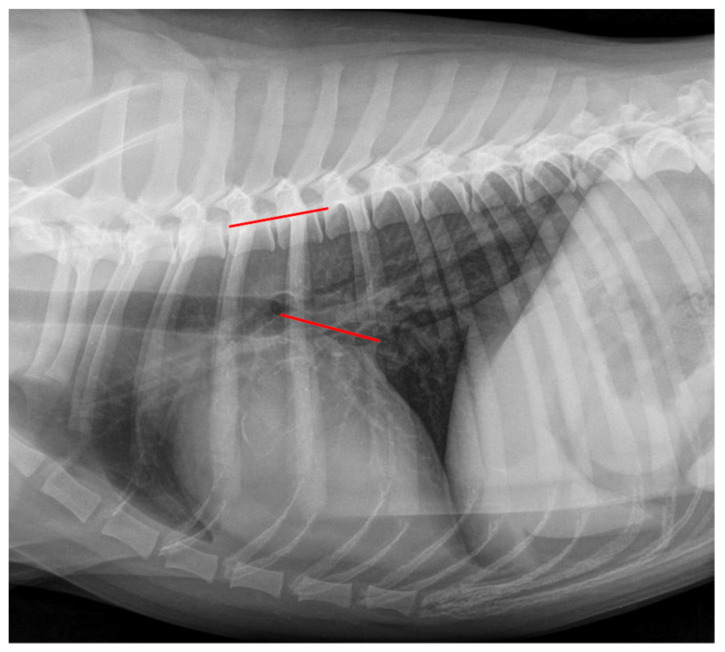
Measurement example of VLAS. The Vertebral Left Atrial Size (VLAS) measurement in the same right lateral thoracic radiograph is shown in Figure 1. A line was drawn from the ventral margin of the carina tracheae to the dorsal intersection between the cardiac silhouette and the caudal vena cava (red line on cardiac silhouette). This line was transposed onto the vertebral column (red line on vertebral) as described in Figure 1. The VLAS was 2.2 vertebral units.

The VLAS was obtained as defined by Malcolm et al. (2018) [23], drawing a line from the ventral margin of the carina tracheae to the dorsal intersection between the cardiac silhouette and the caudal vena cava. Afterward, this line was transposed to the vertebral column and in vertebral units as described previously. A measurement example is displayed in Figure 4.

### 2.5. Observer Grading Assessment

The observer’s confidence during measuring was recorded using a grading system from 1 to 5 developed for this study (Table 1). As a basis for the rating, each observer determined all points of each radiographic measurement, for which they regarded the exact position as difficult to identify. In addition, each observer rated whether they considered the difficulty to have a low or high impact on the radiographic measurement. Then, a difficulty grading for each measurement was assigned, as shown in Table 1. The grading was performed in conventional and inverted radiographs. Each radiographic measurement was graded directly after being obtained.

### 2.6. Statistical Analysis

All statistical analyses were performed using commercial statistical software (SAS-Software, Version 9.4, and SAS Enterprise Guide, version 7.15, SAS Institute Inc., Cary, NC, USA). A *p*-value < 0.05 was considered statistically significant. All measurements were tested for normality using the Shapiro–Wilk omnibus normality test and expressed as mean and standard deviation (SD). The mean radiographic measurements were compared between the different MMVD groups and the healthy control group using a linear mixed model to include correlated results for the same radiograph.

The proposed cut-off values for the radiographic measurements were used. The kappa coefficient compared the reassignment using radiographic measurements in combination with a clinical examination with an echocardiographic assignment.

The intraclass correlation coefficient (ICC) was used to define interobserver and intraobserver variabilities for VHS, RLAD, LA_Width_, and VLAS in conventional and inverted radiographs. For interobserver agreement, a single rating, absolute agreement, and two-way random effects model were applied, and for intraobserver agreement, a single rating, absolute agreement, and two-way fixed effects model were applied. An ICC value of >0.9 was considered almost perfect, 0.75 to 0.9 was considered good, 0.5 to 0.75 was considered moderate, and <0.5 was considered poor.

The observers’ confidence ratings for the measurements and the individual radiographic landmarks were compared in conventional and inverted radiographs, and the results were examined for each observer and group.

## 3. Results

### 3.1. Dogs

A total of 200 dogs (111 males and 89 females) were included in the study. The average age was 10.1 ± 3.3 years (mean ± SD). Dogs in the control group were 6.3 ± 3.0 years old (mean ± SD) and significantly younger compared to the MMVD patients (*p* < 0.0001). The average weight was 15.5 ± 11.5 kg (mean ± SD). Dogs in the control group and B1 group had an average weight of 19.8 ± 11.3 kg and 18.0 ± 11.3 kg (mean ± SD), respectively, and were significantly heavier compared to dogs in the B2 group (12.2 ± 9.4; mean ± SD) and C group (11.9 ± 9.3 kg; mean ± SD) (*p* < 0.0001). The dogs included represented 69 breeds; mixed breed dogs made up the largest group numerically (*n* = 35), followed by Dachshund (*n* = 13), Cavalier King Charles Spaniel (*n* = 9), Chihuahua (*n* = 9), Jack Russell Terrier (*n* = 9), Labrador Retriever (*n* = 9), and Pug (*n* = 8). The breeds were similarly distributed across stages, with some exceptions. For instance, seven Pugs of the eight included in the study were allocated to the control group and only one to stage B1. Furthermore, stages B2 and C included five Dachshunds each. Five of the nine Labrador Retrievers were assigned to the control group and four to stage B1.

### 3.2. Radiographic Measurements—Group Comparison

In total, 1200 radiographic evaluations were performed based on 200 conventional radiographs and 200 corresponding inverted radiographs by three observers. Each radiographic evaluation contained four radiographic measurements. The radiographic measurements taken in conventional radiographs in the different stages by the three observers are displayed in Table 2. All measurements were similar in the control group and stage B1. Patients in stages B2 and C exhibited LA enlargement, confirmed by a significant increase in VHS, RLAD, LA_Width_, and VLAS compared to the previous stage B1 (*p* < 0.01).

The reading of the radiographic measurements in the inverted radiographs is displayed in Supplementary File S4. All measurements were similar in the control group and stage B1. Dogs in stages B2 and C presented a significantly higher VHS, RLAD, LA_Width_, and VLAS compared to the previous stage B1 (*p* < 0.02).

### 3.3. Radiographic Stage Assignment—Comparison of the Different Observers

Using proposed cut-off values in combination with a clinical examination, the radiographic measurements performed by Observers 1 and 2 could reassign approximately 80% of the case dogs to the correct stage. Details and the comparison with an experienced observer (Observer 3) can be found in Table 3.

### 3.4. Comparison between Different Experienced Observers—Difficulty Grading

The observers’ grading regarding the difficulty of assessing the different radiographic measurements is shown in Table 4. Observers graded VHS and VLAS with the best grades (i.e., easiest to measure) and RLAD with the worst in both conventional and inverted radiographs. The grade distribution was similar between Observers 1 and 2, with one exception. Observer 2 graded more radiographs with grade 5 than Observer 1. Observer 3 was most confident in his measurements and awarded grade 1 more often than both other observers. With further disease progression, the observers tended to rate the measurements with a better grade.

### 3.5. Comparison of Different Radiographic Measurements and Landmarks

Evaluating the measurement points in conventional radiographs, VLAS and VHS received the best grading, LA_Width_ a good grading, and RLAD received the worst grading. However, there were better identifiable RLAD measurements when an LA enlargement was present. The landmark, which was the most difficult to detect according to the observers’ rating, was the dorsal margin of the LA. The cranial contour of the cardiac silhouette was the best identifiable landmark. Moreover, the carina tracheae and the ventral intersection point between the cardiac silhouette and vena cava caudalis were also easy to identify in most patients (Table 5). When comparing the different observers’ ratings for the individual landmarks, Observers 1 and 2 evaluated the same amount of measurement difficulties. Besides the dorsal margin of the LA, observer 3 found fewer landmarks difficult to detect (Table 6).

The distribution of measurement difficulties was similar in inverted radiographs compared to conventional radiographs. Thus, the number of difficulties in detecting the dorsal margin of the LA was similar in conventional and inverted radiographs. However, the other landmarks were more difficult to detect in inverted radiographs (Supplementary File S4).

### 3.6. Interobserver Agreement—Radiographic Measuring

The level of agreement among the three observers was almost perfect for VHS measurement, with an ICC of 0.962 (0.948–0.973) in conventional radiographs and an ICC of 0.956 (0.937–0.971) in inverted radiographs. Furthermore, the ICCs remained high in each specific sample group (control group: 0.922, stage B1: 0.894, stage B2: 0.938, stage C: 0.965).

The other three radiographic measurements had a good level of agreement in conventional radiographs, with an ICC of 0.778 for RLAD (0.720–0.826), an ICC of 0.772 for LA_Width_ (0.722–0.886), and an ICC of 0.858 (0.823–0.886) for VLAS. In inverted radiographs, these radiographic measurements also had a good level of agreement, with an ICC of 0.766 for RLAD (0.709–0.814), an ICC of 0.779 for LA_Width_ (0.730–0.819), and an ICC of 0.855 for VLAS (0.820–0.883).

However, based on the individual groups, the ICCs in the control group and stage B1 were lower (RLAD: 0.345 and 0.430, LA_Width_: 0.574 and 0.571, VLAS: 0.544 and 0.655) than in stages B2 and C (RLAD: 0.684 and 0.716, LA_Width_: 0.693 and 0.737, VLAS: 0.788 and 0.871). The distributions of each set of radiographic measurements in conventional radiographs performed by the three different observers are summarized in Supplementary File S4.

### 3.7. Intraobserver Agreement—Radiographic Measuring

A repeated measurement of each observer per case (once in conventional and once in inverted radiographs) showed almost perfect ICC for intraobserver agreement (Table 7).

## 4. Discussions

This study compared the measurement reproducibility of different anatomic landmarks on radiographs for calculating different radiographic measurements, VHS, RLAD, LA_Width_, and VLAS in dogs with and without MMVD. Additionally, obtaining the measurements by the three veterinarians with different levels of experience in evaluating thoracic radiographs was investigated regarding inter- and intraobserver agreement and ease of use in conventional and inverted radiographs.

Less experienced observers can also stage dogs with MMVD using various radiographic measurements in combination with a clinical examination comparable to a specialized veterinarian. Especially the specialized veterinarian assigned more dogs to the correct stage. Nonetheless, when using VHS, the less experienced observers assigned an almost similar number of dogs to the correct stage as a specialized observer (approximately 84%). Using radiographic measurements for the LA size, the experienced observer assigned up to 82% of dogs to the correct stage and less experienced observers up to 79%. In a previous study and in the present study, the specialized veterinarian was superior in staging the dogs according to the measured LA size [36]. However, the staging performed by the less experienced observers shows promising agreement with the echocardiographic examination, thus proving the effective usability of the radiographic measurements. For optimal staging, less experienced observers may use the VHS, and experienced observers can additionally measure the LA size.

Results of this study indicate that VHS, RLAD, LA_Width_, and VLAS are reproducible radiographic measurements that can be utilized by observers with different levels of experience, leading to similar results. Specifically, the ICC of the VHS showed almost perfect results for inter- and intraobserver agreement, which is in line with previous studies [15,29,37,40]. Of the aforementioned studies, only Taylor et al. (2020) repeated all measurements for VHS, but only investigated intraobserver agreement in healthy dogs [40]. The other studies randomly selected up to 20 cases from their study population for inter- and intraobserver agreement [15,29,37]. Only Lam et al. (2021) randomly included dogs suffering from MMVD in their study population [15]. The VHS retains its importance in the radiographic examination of canine cardiac patients as a highly reproducible and commonly used measurement [19,41].

In this study, inter- and intraobserver agreement calculations were based on radiographic measurements of 50 healthy dogs and 150 affected dogs (50 for each of the respective stages, B1, B2, and C). However, it should be considered that the VHS reference ranges appear to vary widely between different breeds of dogs [28,41]. Breed-specific reference values are desirable, and the interpretation in the context of cross-breed values must be made with caution. In addition, the VHS assesses the overall cardiac silhouette rather than the LA size, so other radiographic measurements must be used for LA assessment and detecting a potential LA enlargement due to chronic mitral valve regurgitation in cases of MMVD [2,42].

The VLAS showed a lot of potential in previous studies regarding reproducibility and detection of LA enlargement, confirmed by the good results of the current study on inter- and intraobserver agreement. It must be mentioned that in the present study, the interobserver agreements for the control group and B1 group were worse than in previous studies and only approached previously reported values for stages B2 and C [15,16,23,30,37]. For RLAD and LA_Width_, the interobserver agreement also increased for stages B2 and C. However, the RLAD ICC for the interobserver agreement was lower than previously reported [15,16]. As soon as the LA enlarged, the measurements of different observers were closer together than measurements in radiographs without an enlargement. These radiographic measurements show promising results for LA assessment. Considering the potential observer-related influence, especially in the absence of an LA enlargement, the radiographic measurements should be used additionally for heart assessment.

Regarding comparing the measurements in different disease stages, all measurements generally increased with further disease progression [14,15,16,23,26,30,43,44]. Although there was no significant difference between the control group and stage B1, patients in the control group received slightly higher VHS ratings than those in the B1 group. This may seem surprising at first, but there are two plausible reasons: firstly, a VHS lower than 10.5 is a prerequisite for inclusion in stage B1. This restriction does not apply to dogs in the control group. Secondly, the composition of the two groups regarding breed distribution was not identical, and the VHS can be significantly different in different dog breeds [27,28,40,41].

Regarding the ease of use of the different radiographic measurements, VHS and VLAS received the best results. For VLAS, an important reason may be the low number of radiographic landmarks necessary for obtaining the measurement. With lower landmarks involved, there is less potential for problems occurring when identifying the exact position of the landmarks. In addition, both measurement points were fairly easy to locate, which supports the ease of use of the VLAS. One of the measurement landmarks, the carina trachea, was a part of every measurement and was easy to identify as an ovoid structure above the cardiac silhouette [23]. The ease of finding each landmark in the VHS is related in part to the fact that one of the landmarks applied is the cranial contour of the cardiac silhouette, which was judged to be the easiest of all the points to identify. A reason for that is the air inside the lung provides a contrast to the cardiac silhouette [45]. This contrast seemed to favor the identification of the intersection point between the cardiac silhouette and ventral margin of the vena cava caudalis (measurement point of VHS) more than the dorsal margin of the vena cava caudalis. However, in some cases, the dorsal intersection point could be easily superimposed by ribs, vessel structures, or even the presence of pulmonary edema [16,19]. Starting in the perihilar, a cardiogenic pulmonary edema is mainly located in the caudal lung lobes and increases the opacity of the lung [46]. Surprisingly, the number of assessed radiographs showing difficulties in detecting the dorsal vena cava caudalis did not increase in stage C. The presence of pulmonary edema might be negligible because mild pulmonary edema is usually a subtle radiographic sign [7].

The RLAD was the radiographic measurement presenting the most difficulties. The dorsal margin of the LA was the hardest to identify. Neighboring anatomical structures like pulmonary veins, aorta or ribs reduced visibility of the left atrium and limited the observers’ confidence [16,24]. In cases with LA enlargement, it was easier for the observers to distinguish the dorsal margin of the left atrium from the other structures with soft tissue opacity. However, the three veterinarians still found it difficult in many cases. Additionally, the strict application of the RLAD seemed to limit the measurement. The RLAD line was measured at a 45° angle and could not include the most dorsal bulge of the left atrium in many cases. Considering the irregular LA shape and the complex not uniform LA remodeling [34,47], it can be advantageous to have no strict specification in measuring the most dorsal LA bulge. For measuring the most dorsal LA bulge, Lam et al. (2021) suggested a modified version of the VLAS and showed promising results for detecting a LA enlargement and reproducibility [15].

When comparing the results of three different observers regarding the ease of use, the specialized veterinarian was more confident in measuring and grading the measurement. This result is not surprising [7,10,19]. However, the good interobserver agreement implies that the different radiographic measurements also apply to less experienced observers. All observers found the inverted radiographs more challenging to evaluate than the conventional radiographs. This might be related to conventional radiographs being more commonly evaluated in the daily praxis. Nevertheless, despite the observers’ subjective evaluation, the intraobserver agreement showed almost perfect reproducibility of the various radiographic measurements.

The present study has some limitations. The study population differed significantly in weight, including a broad number of breeds and a low number of individuals in each breed. Some dogs in stages B2 and C received diuretic medication, which could influence the visibility of the cardiac silhouette during pulmonary edema. The recumbency of some dogs was not perfect (slightly rotated or limbs not fully extended), but the quality of the radiographs was appropriate. The observers had different levels of experience, so their measurements were not completely comparable. The rating and the evaluation of the measurement difficulties were strictly subjective. Due to the subsequent measurements, it is possible that the observers were more confident and graded the radiographic measurements better on later radiographs. Additionally, considering the aim of the study, possible factors influencing radiographic measurement results across different stages like breed, size or sex were not examined.

## 5. Conclusions

The radiographic measurements VHS, LA_Width_, and VLAS are easier to measure and showed better interobserver agreement than the RLAD. The VHS is the most reliable and reproducible measurement and shows the most significant agreement with echocardiography. Nevertheless, an echocardiography examination is more accurate. Moreover, the VHS was independent of the observers’ experience and the best radiographic measurement to distinguish between dogs in stages B1 and B2. Despite the excellent intraobserver agreement, a potential observer-dependent influence could be detected for RLAD, LA_Width_, and VLAS.

Considering that VLAS was easier to apply than LA_Width_, VHS and VLAS seem the obvious choices for radiographic evaluation of the canine heart in the future.

The evaluation of the different radiographic landmarks showed that the dorsal margin of the LA was especially challenging, and the cranial margin of the cardiac silhouette was comparatively easy to identify.

In addition to the subjectively more straightforward method of radiographic measurements in conventional radiographs, no differences could be detected between the radiographic methods.

## Figures and Tables

**Table 1:** Explanation of the grading system for the radiographic measurements.

Grade	Meaning
1	The observer was confident in determining the exact position of all points necessary for the radiographic measurement.
2	The observer was uncertain regarding the exact position of one of the measured points, with an expected *low* impact on the final result of the radiographic measurement.
3	The observer was uncertain regarding the exact position of two or more points with an expected *low* impact on the final result of the radiographic measurement.
4	The observer was uncertain regarding the exact position of one of the measured points with an expected *high* impact on the final result of the radiographic measurement.
5	The observer was uncertain regarding the exact position of two or more points with an expected *high* impact on the final result of the radiographic measurement.

**Table 2:** Radiographic measurement among the different observers in conventional radiographs.

		CG	Stage B1	Stage B2	Stage C
VHS	All Observers	10.7 ± 0.8	10.5 ± 0.6 *	11.3 ± 0.8 †	12.2 ± 1.1 ‡
	OB1	10.7 ± 0.9	10.5 ± 0.6 *	11.3 ± 0.8 †	12.1 ± 1.1 ‡
	OB2	10.8 ± 0.8	10.6 ± 0.6 *	11.4 ± 0.8 †	12.3 ± 1.1 ‡
	OB3	10.7 ± 0.8	10.4 ± 0.5 *	11.3 ± 0.7 †	12.2 ± 1.1 ‡
RLAD	All Observers	1.8 ± 0.3	1.8 ± 0.3 *	2.1 ± 0.4 †	2.7 ± 0.5 ‡
	OB1	1.8 ± 0.2	1.9 ± 0.3 *	2.1 ± 0.4	2.6 ± 0.5 ‡
	OB2	1.9 ± 0.3	1.9 ± 0.3 *	2.2 ± 0.4 †	2.7 ± 0.6 ‡
	OB3	1.7 ± 0.3	1.7 ± 0.3	2.1 ± 0.5 †	2.7 ± 0.5 ‡
LA_Width_	All Observers	1.7 ± 0.3	1.7 ± 0.3 *	2.0 ± 0.3 †	2.2 ± 0.3 ‡
	OB1	1.6 ± 0.3	1.7 ± 0.3 *	2.0 ± 0.3 †	2.2 ± 0.3 ‡
	OB2	1.7 ± 0.3	1.7 ± 0.3 *	2.0 ± 0.3 †	2.2 ± 0.3 ‡
	OB3	1.7 ± 0.3	1.6 ± 0.3 *	2.0 ± 0.3 †	2.3 ± 0.4 ‡
VLAS	All Observers	2.1 ± 0.3	2.1 ± 0.3 *	2.5 ± 0.4 †	2.9 ± 0.5 ‡
	OB1	2.0 ± 0.3	2.2 ± 0.3 *	2.5 ± 0.4 †	2.9 ± 0.4 ‡
	OB2	2.2 ± 0.4	2.1 ± 0.3 *	2.5 ± 0.4 †	3.0 ± 0.5 ‡
	OB3	2.1 ± 0.3	2.1 ± 0.3 *	2.5 ± 0.4 †	3.0 ± 0.5 ‡

Radiographic measurements presented as mean ± SD of vertebral units. Staging according to the latest consensus statement of the American Colleague of Veterinary Internal Medicine (ACVIM). Each veterinarian performed the measurements in 50 radiographs per group. The combined measurements of all observers contained 150 measurements. *—Significantly different compared to stages B2 and C (*p* < 0.01). †—significantly different compared to stage B1 (*p* < 0.001). ‡—significantly different compared to stages B1 and B2 (*p* < 0.001). CG—Control group; LA_Width_—Left Atrial Width; OB1—recently graduated veterinarian; OB2—experienced veterinarian; OB3—specialized veterinarian; RLAD—Radiographic Left Atrial Dimension; VHS—Vertebral Heart Size; VLAS—Vertebral Atrial Size.

**Table 3:** Comparison of radiographic reassigning of the dogs with the echocardiographic assignment. Supplementary File S3.

		Conventional Radiographs			Inverted Radiographs		
Radiographic measurement	Cut-off values	OB1	OB2	OB3 *	OB1	OB2	OB3
VHS	11.0 *	0.840	0.833	0.847	0.833	0.827	0.853 *
RLAD	2.0 *	0.767	0.747	0.807	0.793	0.767	0.820 *
LA_Width_	1.8 *	0.793	0.793	0.813	0.753	0.800	0.847 *
VLAS	2.3 *	0.787	0.793	0.820	0.793	0.780	0.833 *

LA_Width_—Left Atrial Width; OB1—recently graduated veterinarian; OB2—experienced veterinarian; OB3—specialized veterinarian; RLAD—Radiographic Left Atrial Dimension; VHS—Vertebral Heart Size; VLAS—Vertebral Atrial Size. Reassignments of the dogs using radiographic cut-off values and a clinical examination. The kappa coefficient compared this reassignment with the echocardiographic assignment according to the latest consensus statement of the American Colleague of Veterinary Internal Medicine (ACVIM). For the radiographic assignment, proposed cut-off values were used. *—data from Levicar et al., 2022 [17].

**Table 4:** Grading of each measurement in conventional radiographs.

Grades		CG			Stage B1			Stage B2			Stage C		
		OB1	OB2	OB3	OB1	OB2	OB3	OB1	OB2	OB3	OB1	OB2	OB3
VHS	1	17	21	40	22	25	42	23	24	45	13	16	40
	2	23	18	5	19	14	6	19	17	3	24	19	5
	3	4	-	3	4	2	1	5	1	1	4	1	2
	4	2	1	1	3	1	-	2	-	1	3	1	2
	5	4	10	1	2	8	1	1	8	-	6	13	1
RLAD	1	2	3	3	3	4	6	7	3	10	2	3	21
	2	7	8	14	11	13	14	14	10	15	18	13	13
	3	11	3	2	13	3	5	12	2	1	10	3	3
	4	7	7	17	7	6	15	7	9	19	6	1	7
	5	23	29	14	16	24	10	10	26	5	14	30	6
LA_Width_	1	12	17	34	16	20	35	22	19	43	8	15	39
	2	20	16	10	20	11	11	19	18	3	29	21	4
	3	6	1	3	5	5	-	5	1	2	2	-	2
	4	6	-	1	4	3	3	2	2	2	5	3	5
	5	6	16	2	5	11	1	2	10	-	6	11	-
VLAS	1	27	23	35	26	23	36	35	29	44	31	30	44
	2	11	17	11	15	19	10	10	13	3	10	10	-
	3	-	1	1	1	-	-	1	-	1	-	-	2
	4	12	4	2	7	5	3	4	2	2	7	7	4
	5	-	5	1	1	3	1	-	6	-	2	3	-

Summarized observers’ confidence in the radiographic measurements. Three veterinarians with different levels of experience level (recently graduated veterinarian, experienced veterinarian, and specialized veterinarian) performed the grading. Grades represented the difficulty in recognizing the anatomical landmarks and defining the radiographic measurements. Staging according to the latest consensus statement of the American Colleague of Veterinary Internal Medicine (ACVIM). CG—Control group; LA_Width_—Left Atrial Width; OB1—recently graduated veterinarian; OB2—experienced veterinarian; OB3—specialized veterinarian; RLAD—Radiographic Left Atrial Dimension; VHS—Vertebral Heart Size; VLAS—Vertebral Atrial Size.

**Table 5:** Distribution of conventional well identifiable radiographs and radiographs with measurement difficulties in the different groups.

Evaluation Criteria	All Groups *n* = 600 (%)	CG *n* = 150 (%)	Stage B1 *n* = 150 (%)	Stage B2 *n* = 150 (%)	Stage C *n* = 150 (%)
Well identifiable radiographs					
VHS	328 (54.7)	78 (52)	89 (59.3)	92 (61.3)	69 (46)
RLAD	67 (11.2)	8 (5.3)	13 (8.7)	20 (13.3)	26 (17.3)
LA_Width_	281 (46.8)	63 (42)	71 (47.3)	85 (56.7)	62 (41.3)
VLAS	383 (63.8)	85 (56.7)	85 (56.7)	108 (72)	105 (70)
radiographic landmarks					
carina tracheae *,**,†, ‡	79 (13.2)	27 (18)	18 (12)	18 (12)	16 (10.7)
dorsal margin of left atrium **	480 (80)	135 (90)	128 (85.3)	120 (80)	97 (64.7)
dorsal vena cava caudalis **,†, ‡	168 (25)	46 (30.7)	53 (35.3)	32 (21.3)	37 (24.7)
ventral vena cava caudalis *	76 (12.7)	15 (10)	18 (12)	15 (10)	28 (18.7)
apex cordis *,**,†	199 (33.2)	53 (35.3)	40 (26.7)	41 (27.3)	65 (43.3)
cranial heart side *	16 (2.7)	2 (1.3)	5 (3.3)	3 (2)	6 (4)

Three different veterinarians performed 600 radiographic evaluations (50 per group and 200 per observer) with different levels of experience (recently graduated veterinarian, experienced veterinarian, and specialized veterinarian); multiple selections are possible. Observers rated the same radiographs. Staging according to the latest consensus statement of the American Colleague of Veterinary Internal Medicine (ACVIM). *—part of VHS measurement. **—part of RLAD measurement. †—part of LA_Width_ measurement. ‡—part of VLAS measurement. CG—Control group; LA_Width_—Left Atrial Width; RLAD—Radiographic Left Atrial Dimension; VHS—Vertebral Heart Size; VLAS—Vertebral Atrial Size.

**Table 6:** Distribution of conventional well identifiable radiographs and radiographs with measurement difficulties measured by the individual observers.

Evaluation Criteria	CG			Stage B1			Stage B2			Stage C		
	OB1	OB2	OB3	OB1	OB2	OB3	OB1	OB2	OB3	OB1	OB2	OB3
Well identifiable radiographs												
VHS	17	21	40	22	25	42	23	24	45	12	16	41
RLAD	2	3	3	3	4	6	7	3	10	2	3	21
LA_Width_	2	17	34	16	20	35	22	20	43	8	15	39
VLAS	27	23	35	26	23	36	35	29	44	31	30	40
radiographic landmarks												
carina tracheae *,**,†, ‡	7	12	8	6	7	5	6	8	4	5	8	3
dorsal margin of left atrium **	44	45	46	41	43	44	36	45	39	30	42	25
dorsal vena cava caudalis **,†, ‡	16	21	9	20	23	10	10	19	3	16	16	5
ventral vena cava caudalis *	6	7	2	7	7	4	6	9	0	11	13	4
apex cordis *,**,†	27	22	4	19	19	2	20	19	2	32	27	6
cranial heart side *	1	1	0	2	2	1	2	1	0	1	4	1

Three different veterinarians performed 600 radiographic evaluations (50 per group and 200 per observer) with different levels of experience (OB 1, OB 2, and OB 3); multiple selections were possible. Observers rated the same radiographs. Staging according to the latest consensus statement of the American Colleague of Veterinary Internal Medicine (ACVIM). *—part of VHS measurement. **—part of RLAD measurement. †—part of LA_Width_ measurement. ‡—part of VLAS measurement. CG—Control group; LA_Width_—Left Atrial Width; OB1—recently graduated veterinarian; OB2—experienced veterinarian; OB3—specialized veterinarian; RLAD—Radiographic Left Atrial Dimension; VHS—Vertebral Heart Size; VLAS—Vertebral Atrial Size.

**Table 7:** Intraobserver agreement of each observer obtaining the radiographic measurements in conventional and inverted radiographs.

	VHS	RLAD	LA_Width_	VLAS
OB1	0.985 (0.981–0.989)	0.929 (0.908–0.946)	0.910 (0.883–0.931)	0.944 (0.928–0.958)
OB2	0.978 (0.971–0.983)	0.920 (0.896–0.938)	0.926 (0.903–0.943)	0.929 (0.904–0.943)
OB3	0.983 (0.978–0.987)	0.943 (0.926–0.987)	0.924 (0.901–0.941)	0.964 (0.953–0.972)

Intraclass correlation coefficient (ICC) between the different radiographic measurements measured by each observer in conventional and inverted radiographs. LA_Width_—Left Atrial Width; OB1—recently graduated veterinarian; OB2—experienced veterinarian; OB3—specialized veterinarian; RLAD—Radiographic Left Atrial Dimension; VHS—Vertebral Heart Size; VLAS—Vertebral Atrial Size.

## Data Availability

The data presented in this study are available on request from the corresponding author.

## Supplementary Materials

The following supporting information can be downloaded at: https://www.mdpi.com/article/10.3390/ani12192531/s1, Word document Supplementary File S1: Table for echocardiographic data and medication, Supplementary File S2: Table for radiographic settings, Supplementary File S3: Measurement examples in inverted radiographs and graphic displaying measurements performed by all three observers, Supplementary File S4: Tables for inverted radiographs.

## References

[1] Martin M.W.S., Stafford Johnson M.J., Celona B. (2009). Canine dilated cardiomyopathy: A retrospective study of signalment, presentation and clinical findings in 369 cases. J. Small Anim. Pract..

[2] Borgarelli M., Haggstrom J. (2010). Canine degenerative myxomatous mitral valve disease: Natural history, clinical presentation and therapy. Vet. Clin. N. Am. Small Anim. Pract..

[3] Keene B.W., Atkins C.E., Bonagura J.D., Fox P.R., Haggstrom J., Fuentes V.L., Oyama M.A., Rush J.E., Stepien R., Uechi M. (2019). ACVIM consensus guidelines for the diagnosis and treatment of myxomatous mitral valve disease in dogs. J. Vet. Intern. Med..

[4] Kresken J.G., Wendt R., Molder P. (2019). Praxis der Kardiologie.

[5] Meomartino L., Greco A., Di Giancamillo M., Brunetti A., Gnudi G. (2021). Imaging techniques in Veterinary Medicine. Part I: Radiography and Ultrasonography. Eur. J. Radiol. Open.

[6] Wesselowski S., Gordon S.G., Meddaugh N., Saunders A.B., Haggstrom J., Cusack K., Janacek B., Matthews D. (2022). Prediction of clinically important acquired cardiac disease without an echocardiogram in large breed dogs using a combination of clinical, radiographic and electrocardiographic variables. J. Vet. Cardiol..

[7] Hansson K., Haggstrom J., Kvart C., Lord P. (2009). Reader performance in radiographic diagnosis of signs of mitral regurgitation in cavalier King Charles spaniels. J. Small Anim. Pract..

[8] Guglielmini C., Diana A. (2015). Thoracic radiography in the cat: Identification of cardiomegaly and congestive heart failure. J. Vet. Cardiol..

[9] Mostafa A.A., Berry C.R. (2017). Radiographic assessment of the cardiac silhouette in clinically normal large- and small-breed dogs. Am. J. Vet. Res..

[10] Duler L., LeBlanc N.L., Cooley S., Nemanic S., Scollan K.F. (2018). Interreader agreement of radiographic left atrial enlargement in dogs and comparison to echocardiographic left atrial assessment. J. Vet. Cardiol..

[11] Killich K. (2018). Kleintierkardiologie.

[12] Atkins C., Bonagura J., Ettinger S., Fox P., Gordon S., Haggstrom J., Hamlin R., Keene B., Fuentes V.L., Stepien R. (2009). Guidelines for the Diagnosis and Treatment of Canine Chronic Valvular Heart Disease. J. Vet. Intern. Med..

[13] Chetboul V., Tissier R. (2012). Echocardiographic assessment of canine degenerative mitral valve disease. J. Vet. Cardiol..

[14] Kim S., Seo K.W., Song K.-H. (2020). An Assessment of Vertebral Left Atrial Size in Relation to the Progress of Myxomatous Mitral Valve Disease in Dogs. J. Vet. Clin..

[15] Lam C., Gavaghan B.J., Meyers F.E. (2021). Radiographic quantification of left atrial size in dogs with myxomatous mitral valve disease. J. Vet. Intern. Med..

[16] Vezzosi T., Puccinelli C., Citi S., Tognetti R. (2021). Two radiographic methods for assessing left atrial enlargement and cardiac remodeling in dogs with myxomatous mitral valve disease. J. Vet. Cardiol..

[17] Levicar C., Granados-Soler J.L., Freise F., Raue J.F., Nolte I., Bach J.-P. (2022). Comparison of different radiographic scores with associated echocardiographic measurements and prediction of heart enlargement in dogs with and without myxomatous mitral valve disease. J. Vet. Cardiol..

[18] Boswood A., Haggstrom J., Gordon S.G., Wess G., Stepien R.L., Oyama M.A., Keene B.W., Bonagura J., MacDonald K.A., Patteson M. (2016). Effect of Pimobendan in Dogs with Preclinical Myxomatous Mitral Valve Disease and Cardiomegaly: The EPIC Study-A Randomized Clinical Trial. J. Vet. Intern. Med..

[19] Hansson K., Haggstrom J., Kvart C., Lord P. (2005). Interobserver variability of vertebral heart size measurements in dogs with normal and enlarged hearts. Vet. Radiol. Ultrasound.

[20] Buchanan J.W., Bücheler J. (1995). Vertebral scale system to measure canine heart size in radiographs. J. Am. Vet. Med. Assoc..

[21] Le Roux A., Rademacher N., Saelinger C., Rodriguez D., Pariaut R., Gaschen L. (2012). Value of tracheal bifurcation angle measurement as a radiographic sign of left atrial enlargement in dogs. Vet. Radiol. Ultrasound.

[22] Torad F.A., Hassan E.A. (2014). Two-dimensional cardiothoracic ratio for evaluation of cardiac size in German shepherd dogs. J. Vet. Cardiol..

[23] Malcolm E.L., Visser L.C., Phillips K.L., Johnson L.R. (2018). Diagnostic value of vertebral left atrial size as determined from thoracic radiographs for assessment of left atrial size in dogs with myxomatous mitral valve disease. J. Am. Vet. Med. Assoc..

[24] Sanchez Salguero X., Prandi D., Llabres-Diaz F., Manzanilla E.G., Bussadori C. (2018). A radiographic measurement of left atrial size in dogs. Ir. Vet. J..

[25] Sanchez Salguero X., Prandi D., Llabrés-Díaz F., Manzanilla E.G., Badiella L., Bussadori C. (2019). Heart to spine measurements to detect left atrial enlargement in dogs with mitral insufficiency. Ir. Vet. J..

[26] Stepien R.L., Rak M.B., Blume L.M. (2020). Use of radiographic measurements to diagnose stage B2 preclinical myxomatous mitral valve disease in dogs. J. Am. Vet. Med. Assoc..

[27] Kraetschmer S., Ludwig K., Meneses F., Nolte I., Simon D. (2008). Vertebral heart scale in the beagle dog. J. Small Anim. Pract..

[28] Jepsen-Grant K., Pollard R.E., Johnson L.R. (2013). Vertebral heart scores in eight dog breeds. Vet. Radiol. Ultrasound.

[29] Vezzosi T., Puccinelli C., Tognetti R., Pelligra T., Citi S. (2020). Radiographic vertebral left atrial size: A reference interval study in healthy adult dogs. Vet. Radiol. Ultrasound.

[30] Poad M.H., Manzi T.J., Oyama M.A., Gelzer A.R. (2020). Utility of radiographic measurements to predict echocardiographic left heart enlargement in dogs with preclinical myxomatous mitral valve disease. J. Vet. Intern. Med..

[31] Barr F., Birch S., Kirberger R.M., McEvoy F.J. (2016). Soft tissues. BSAVA Manual of Canine and Feline Musculoskeletal Imaging.

[32] Kirchner J., Gadek D., Goltz J.P., Doroch-Gadek A., Stückradt S., Liermann D., Kickuth R. (2013). Standard versus inverted digital luminescence radiography in detecting pulmonary nodules: A ROC analysis. Eur. J. Radiol..

[33] Cheng C.-J., Mandour A.S., Yoshida T., Watari T., Tanaka R., Matsuura K. (2022). Changes in renin-angiotensin-aldosterone system during cardiac remodeling after mitral valvuloplasty in dogs. J. Vet. Intern. Med..

[34] Nakayama H., Nakayama T., Hamlin R.L. (2001). Correlation of cardiac enlargement as assessed by vertebral heart size and echocardiographic and electrocardiographic findings in dogs with evolving cardiomegaly due to rapid ventricular pacing. J. Vet. Intern. Med..

[35] Saida Y., Tanaka R., Yamane Y., Suzuki K., Maruyama R., Koie H., Matsumoto T., Asano R. (2006). Relationships between Vertebral Heart Size (VHS) and Echocardiographic Parameters in Dogs with Mitral Regurgitation. Adv. Anim. Cardiol..

[36] Bagardi M., Manfredi M., Zani D.D., Brambilla P.G., Locatelli C. (2021). Interobserver variability of radiographic methods for the evaluation of left atrial size in dogs. Vet. Radiol. Ultrasound.

[37] Baisan R.A., Vulpe V. (2021). Vertebral heart size and vertebral left atrial size reference ranges in healthy Maltese dogs. Vet. Radiol. Ultrasound.

[38] Hansson K., Haggstrom J., Kvart C., Lord P. (2002). Left atrial to aortic root indices using two-dimensional and M-mode echocardiography in cavalier King Charles spaniels with and without left atrial enlargement. Vet. Radiol. Ultrasound.

[39] Cornell C.C., Kittleson M.D., Della Torre P., Haggstrom J., Lombard C.W., Pedersen H.D., Vollmar A., Wey A. (2004). Allometric scaling of M-mode cardiac measurements in normal adult dogs. J. Vet. Intern. Med..

[40] Taylor C.J., Simon B.T., Stanley B.J., Lai G.P., Thieman Mankin K.M. (2020). Norwich terriers possess a greater vertebral heart scale than the canine reference value. Vet. Radiol. Ultrasound.

[41] Lamb C., Wikeley H., Boswood A., Pfeiffer D. (2001). Use of breed-specific ranges for vertebral heart scale in dogs as an aid to radiographic diagnosis of cardiac disease. Vet. Rec..

[42] Borgarelli M., Crosara S., Lamb K., Savarino P., La Rosa G., Tarducci A., Haggstrom J. (2012). Survival characteristics and prognostic variables of dogs with preclinical chronic degenerative mitral valve disease attributable to myxomatous degeneration. J. Vet. Intern. Med..

[43] Lord P., Hansson K., Kvart C., Haggstrom J. (2010). Rate of change of heart size before congestive heart failure in dogs with mitral regurgitation. J. Small Anim. Pract..

[44] Lord P.F., Hansson K., Carnabuci C., Kvart C., Haggstrom J. (2011). Radiographic heart size and its rate of increase as tests for onset of congestive heart failure in Cavalier King Charles Spaniels with mitral valve regurgitation. J. Vet. Intern. Med..

[45] Andrei B., Birsan O., Vulpe V. (2016). The diagnostic value of cardio-thoracic ratio in detecting heart size changes in dog. Rev. Rom. Med. Vet..

[46] Diana A., Guglielmini C., Pivetta M., Sanacore A., Di Tommaso M., Lord P.F., Cipone M. (2009). Radiographic features of cardiogenic pulmonary edema in dogs with mitral regurgitation: 61 cases (1998–2007). J. Am. Vet. Med. Assoc..

[47] Goette A., Kalman J.M., Aguinaga L., Akar J., Cabrera J.A., Chen S.A., Chugh S.S., Corradi D., D’Avila A., Dobrev D. (2016). EHRA/HRS/APHRS/SOLAECE expert consensus on atrial cardiomyopathies: Definition, characterization, and clinical implication. Europace.

