# Correction: Levicar et al. Methods of Radiographic Measurements of Heart and Left Atrial Size in Dogs with and without Myxomatous Mitral Valve Disease: Intra- and Interobserver Agreement and Practicability of Different Methods. *Animals* 2022, *12,* 2531

**DOI:** 10.3390/ani15081082

**Published:** 2025-04-09

**Authors:** Charanthorn Levicar, Ingo Nolte, José Luis Granados-Soler, Fritjof Freise, Jonathan Friedemann Raue, Jan-Peter Bach

**Affiliations:** 1 Clinic for Small Animals, University of Veterinary Medicine Hannover, Foundation, 30559 Hannover, Germany; 2 Institute for Biometry, Epidemiology and Information Processing, University of Veterinary Medicine Hannover, Foundation, 30559 Hannover, Germany

## Error in Figure 2

In the original publication by Levicar et al. (2022) [1], there was a mistake in the legend for Figure 2. Measurement example of RLAD. The RLAD line was too long. The correct legend appears below. Charanthorn Levicar's email address has changed to charanthorn.levicar@web.de. The authors state that the scientific conclusions are unaffected. This correction was approved by the Academic Editor. The original publication has also been updated.

**Figure 2:**
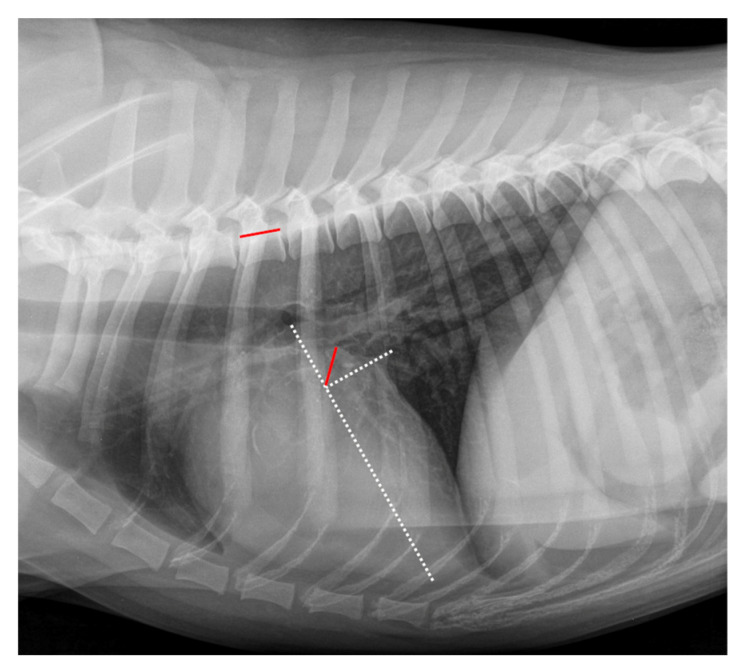
Radiographic Left Atrial Dimension (RLAD) measurement in the same right lateral thoracic radiograph seen in Figure 1. The long axis (white dotted line) was applied as described for the VHS measurement (Figure 1). The short axis (white dotted line) was drawn from dorsal intersection of the caudal vena cava and the cardiac silhouette to the long axis. The bisecting RLAD line was drawn from the intersection point to the dorsal margin of the left atrium (red line on cardiac silhouette). This line was transposed onto the vertebral column (red line on vertebral) as described in Figure 1. The RLAD was 0.8 vertebral units.
