# Supplementary material for: Methods of Radiographic Measurements of Heart and Left Atrial Size in Dogs with and without Myxomatous Mitral Valve Disease: Intra- and Interobserver Agreement and Practicability of Different Methods
Source: Animals (Basel). 2022 Sep 22;12(19):2531. doi: 10.3390/ani12192531 (PMC9559670; doi:10.3390/ani12192531)
Supplement: Supplementary file 1 [file animals-12-02531-s001.zip › Supplementary File S1.pdf]

Additional Table a. Echocardiographic values and medication of examined dogs

CG		Stage B1		Stage B2			Stage C		
LA/Ao	LVIDDn	LA/Ao	LVIDDn	LA/Ao	LVIDDn	medication	LA/Ao	LVIDDn	medication
1.34	1.37	1.2	1.3	1.9	1.9	Pimo, Diur, ACE	2.2	1.7	no
1.3	1.03	1.0	1.56	1.9	2.0	no	1.65	2.1	Pimo
1.31	1.41	0.86	1.3	1.9	1.9	no	3.5	2.6	Diur, ACE
1.43	1.3	1.4	1.36	1.9	1.76	ACE	2.59	2.32	no
1.36	1.28	1.3	1.4	2.0	2.34	Pimo	3.0	2.34	Pimo, Diur, ACE
1.32	1.44	1.24	1.5	1.7	1.95	no	1.7	2.1	Pimo, Diur, ACE
1.25	1.25	1.4	1.53	1.9	1.78	no	1.8	2.1	no
1.0	1.38	1.15	1.26	1.72	2	no	2.6	2.3	no
1.16	1.48	1.48	1.5	1.8	2.27	no	2.6	1.89	Pimo, Diur
1.4	1.49	1.2	1.2	2.0	2.2	no	2.2	1.87	Pimo, Diur, ACE
1.32	1.5	1.4	1.56	2.1	1.97	Pimo	2.9	2.1	Pimo
1.16	1.34	1.22	1.6	1.85	1.87	Pimo, ACE	1.9	1.75	Pimo, Diur
1.1	1.04	1.4	1.34	1.7	1.77	Pimo	1.8	2.16	Pimo, Diur, ACE
1.2	1.46	1.4	1.52	2.25	2.24	no	1.8	2.1	ACE
1.1	1.5	1.2	1.4	1.89	1.8	no	2.3	2.0	Pimo, Diur, ACE
1.16	1.4	1.4	1.4	1.7	2.0	no	2.5	2.1	Diur, ACE
1.2	1.3	1.23	1.51	2.0	1.89	Pimo, ACE	2.2	2.0	ACE
1.3	1.28	1.3	1.1	1.85	2.0	ACE	1.8	2.2	no
1.0	1.4	1.0	1.63	1.75	1.78	no	2.0	2.0	no
1.4	1.49	1.0	1.4	1.75	1.91	no	2.0	2.0	Pimo, Diur, ACE
1.22	1.54	1.5	1.45	1.7	1.86	no	3.1	2.5	Pimo, Diur, ACE
1.24	1.3	1.04	1.51	1.7	1.7	no	2.4	1.83	no
1.2	1.3	1.1	1.48	1.7	1.83	no	2.5	2.3	Pimo, Diur, ACE
1.2	1.5	0.9	1.46	1.9	2.01	Pimo	2.6	2.48	Pimo, Diur, ACE
1.4	1.6	1.5	1.38	1.8	1.72	no	2.0	1.84	no
1.0	1.3	1.24	1.35	1.9	1.93	no	2.7	2.1	Pimo, Diur

1.2	1.3	1.1	1.31	1.9	1.97	no	2.33	2.7	no
1.25	1.3	1.2	1.53	1.8	1.79	Diur, ACE	2.9	1.8	no
1.5	1.4	1.1	1.43	2.0	1.82	Pimo, Diur, ACE	1.9	1.9	Diur, ACE
1.27	1.35	1.5	1.64	1.82	1.73	Pimo, Diur, ACE	2.58	2.44	Pimo, Diur, ACE
1.07	1.6	1.3	1.26	1.9	2.0	Pimo, Diur, ACE	2.8	1.89	Diur, ACE
1.1	1.47	1.35	1.45	1.95	2.08	Pimo	1.8	2.0	Pimo, Diur, ACE
1.46	1.3	1.21	1.2	1.9	1.95	no	1.8	2.29	no
1.21	1.31	1.09	1.42	1.7	1.7	Pimo, Diur, ACE	1.8	1.86	no
0.82	1.35	1.15	1.57	1.8	2.0	no	2.21	2.31	no
1.23	1.31	1.39	1.4	2.0	2.03	Pimo, ACE	2.2	2.26	no
1.13	1.6	1.3	1.46	1.85	1.8	no	1.65	2.01	Pimo, Diur, ACE
1.25	1.03	1.27	1.39	1.8	2.15	Pimo, ACE	2.89	1.9	Pimo, Diur, ACE
1.47	1.35	1.15	1.54	2.0	1.93	no	2.8	2.3	Pimo, Diur, ACE
1.4	1.25	1.46	1.43	1.8	2.1	Pimo, Diur, ACE	2.0	1.98	Diur, ACE
0.96	1.1	1.34	1.34	2	2.3	Diur, ACE	2.6	2.33	Pimo, Diur, ACE
1.21	1.4	1.35	1.58	2.1	1.94	Diur, ACE	2.2	1.94	Pimo, Diur, ACE
0.87	1.3	1.39	1.4	1.84	2.07	no	2.1	2.2	Diur, ACE
1.5	1.4	1.4	1.38	1.9	2.28	no	3.1	2.25	Pimo, Diur, ACE
1.25	1.3	1.4	1.4	1.7	1.74	Diur, ACE	2.5	2.1	Pimo, Diur, ACE
1.41	1.4	1.2	1.2	1.61	1.98	Pimo	2.8	2.28	Diur, ACE
1.54	1.4	1.2	1.4	1.82	1.76	no	2.8	2.19	Diur, ACE
1.29	1.1	1.2	1.57	2.1	2.0	no	2.86	2.4	Pimo, Diur, ACE
1.3	1.4	1.3	1.5	1.9	2.4	no	3.09	1.9	no
1.4	1.4	1.1	1.51	1.6	1.87	no	2.36	1.97	Pimo, Diur, ACE

Staging according to the American College of Veterinary Internal Medicine (ACVIM). Dogs allocated to the control group and stage B1 received no medication. 22 dogs allocated to stage B2 and 36 dogs allocated to stage c reveived medication prior echocardiographic and radiographic examination. Medication included Pimobendan (Pimo), diuretics (Diur) and ACE-inhibitors (ACE).

CG: Control group; LA/Ao: left atrium-to-aorta ratio; LVIDDn: normalized left ventricular internal dimension at end diastole
