# Supplementary material for: Methods of Radiographic Measurements of Heart and Left Atrial Size in Dogs with and without Myxomatous Mitral Valve Disease: Intra- and Interobserver Agreement and Practicability of Different Methods
Source: Animals (Basel). 2022 Sep 22;12(19):2531. doi: 10.3390/ani12192531 (PMC9559670; doi:10.3390/ani12192531)
Supplement: Supplementary file 1 [file animals-12-02531-s001.zip › Supplementary File S2.pdf]

Additional Table b. Radiographic settings

Weight (kg)	0-2	3-5	6-10	11-15	16-20	21-30	31-40	41-50	51-60	61-70	>71
lateral thoracic view	52/8	55/8	60/8	60/8	70/8	70/8	73/8	73/8	73/8	77/8	81/10

Tube voltage and miliampereseconds are indicated (kV/mAs).
