# Supplementary material for: Methods of Radiographic Measurements of Heart and Left Atrial Size in Dogs with and without Myxomatous Mitral Valve Disease: Intra- and Interobserver Agreement and Practicability of Different Methods
Source: Animals (Basel). 2022 Sep 22;12(19):2531. doi: 10.3390/ani12192531 (PMC9559670; doi:10.3390/ani12192531)
Supplement: Supplementary file 1 [file animals-12-02531-s001.zip › Supplementary File S3.pdf]

Additional Table c. Radiographic measurements among the different observers in inverted radiographs

		CG	Stage B1	Stage B2	Stage C
VHS	All Observers	10.7 ± 0.8*	10.5 ± 0.6	11.3 ± 0.8**	12.2 ± 1.1***
	OB1	10.7 ± 0.9*	10.4 ± 0.6	11.3 ± 0.9**	12.1 ± 1.1***
	OB2	10.9 ± 0.8	10.6 ± 0.6	11.4 ± 0.7**	12.3 ± 1.0***
	OB3	10.7 ± 0.8*	10.4 ± 0.5	11.3 ± 0.8**	12.1 ± 1.1***
RLAD	All Observers	1.8 ± 0.3*	1.8 ± 0.3	2.1 ± 0.4**	2.6 ± 0.5***
	OB1	1.7 ± 0.3*	1.8 ± 0.3	2.1 ± 0.4**	2.6 ± 0.5***
	OB2	1.9 ± 0.3	1.8 ± 0.3	2.1 ± 0.5**	2.6 ± 0.5***
	OB3	1.6 ± 0.3*	1.6 ± 0.3	2.1 ± 0.5**	2.7 ± 0.5***
LA_{Width}	All Observers	1.7 ± 0.3*	1.7 ± 0.3	2.0 ± 0.4**	2.2 ± 0.4***
	OB1	1.6 ± 0.3*	1.7 ± 0.3	1.9 ± 0.3**	2.2 ± 0.3***
	OB2	1.7 ± 0.3*	1.6 ± 0.3	1.9 ± 0.4**	2.2 ± 0.4***
	OB3	1.7 ± 0.3*	1.6 ± 0.3	2.0 ± 0.3**	2.3 ± 0.4***
VLAS	All Observers	2.1 ± 0.3*	2.1 ± 0.3	2.5 ± 0.4**	3.0 ± 0.5***
	OB1	2.1 ± 0.3*	2.2 ± 0.3	2.5 ± 0.4**	2.9 ± 0.4***
	OB2	2.2 ± 0.4*	2.1 ± 0.3	2.5 ± 0.5**	3.0 ± 0.5***
	OB3	2.0 ± 0.3*	2.1 ± 0.3	2.5 ± 0.4**	3.0 ± 0.4***

Radiographic measurements presented as mean ± SD of vertebral units. Staging according to the latest consensus statement of the American Colleague of Veterinary Internal Medicine (ACVIM). Each veterinarian performed the measurements in 50 radiographs per group. The combined measurements of all observers contained 150 measurements.

*: significantly different compared to stage B2 and C ($p < 0.002$).

**: significantly different compared to stage B1 ($p < 0.03$).

***: significantly different compared to stage B1 and B2 ($p < 0.02$).

CG: Control group; LA_{Width}: Left Atrial Width; OB1: recently graduated veterinarian; OB2: experienced veterinarian; OB3: specialized veterinarian; RLAD: Radiographic Left Atrial Dimension; VHS: Vertebral Heart Size; VLAS: Vertebral Atrial Size.

Additional Table d. Grading of each radiographic measurement in inverted radiographs

Grades for radiographic measurement		Control group			Stage B1			Stage B2			Stage C		
		OB1	OB2	OB3	OB1	OB2	OB3	OB1	OB2	OB3	OB1	OB2	OB3
VHS	1	12	13	27	22	18	27	13	18	31	12	10	27
	2	19	17	13	13	18	13	25	14	7	24	20	11
	3	8	-	4	3	2	3	2	3	3	6	1	1
	4	3	1	2	6	-	5	7	1	5	2	-	2
	5	8	19	4	6	12	2	3	14	4	6	19	9
RLAD	1	2	2	2	3	3	4	6	3	2	-	1	10
	2	6	3	6	10	10	3	13	6	13	21	9	9
	3	10	-	1	11	4	4	9	4	1	13	4	2
	4	4	6	17	7	7	20	4	6	14	5	1	12
	5	28	39	24	19	26	19	18	31	20	11	35	17
LA_{Width}	1	8	10	20	13	15	26	12	11	29	8	7	29
	2	16	10	19	21	11	10	21	18	6	28	19	4
	3	8	2	-	4	8	2	6	3	1	4	3	2
	4	7	2	6	6	2	8	8	2	7	3	-	4
	5	11	26	5	6	14	4	3	16	7	7	21	11
VLAS	1	20	13	26	25	19	27	29	18	30	30	18	32
	2	11	20	13	14	20	11	11	22	6	10	15	3
	3	2	-	-	1	1	1	-	-	-	1	-	1
	4	13	7	7	9	2	7	9	2	9	8	8	6
	5	4	10	4	1	8	4	1	8	5	1	9	8

Summarized observers' confidence for the radiographic measurements. Three veterinarians with different levels of experience (recently graduated veterinarian, experienced veterinarian, and specialized veterinarian) performed the grading. Grades represented the difficulty in recognizing the anatomical landmarks and defining the radiographic measurements. Staging according to latest consensus statement of the American Colleague of Veterinary Internal Medicine (ACVIM).

LA_{Width}: Left Atrial Width; OB1: recently graduated veterinarian; OB2: experienced veterinarian; OB3: specialized veterinarian; RLAD: Radiographic Left Atrial Dimension; VHS: Vertebral Heart Size; VLAS: Vertebral Atrial Size.

Additional Table 3. Distribution of inverted well-identifiable radiographs and radiographs with measurement difficulties in the different groups

Evaluation criteria	all groups n=600 (%)	CG n=150 (%)	Stage B1 n=150 (%)	Stage B2 n=150 (%)	Stage C n=150 (%)
Well-identifiable radiographs					
VHS	232 (38.7)	52 (34,7)	67 (44.7)	64 (42.7)	49 (32.7)
RLAD	38 (6.3)	6 (4)	10 (6.6)	11 (7.3)	11 (7.3)
LA_{Width}	190 (31.7)	39 (26)	54 (36)	53 (35.3)	44 (29.3)
VLAS	287 (47.8)	59 (39.3)	71 (47.3)	77 (51.3)	80 (53.3)
anatomical landmarks					
carina tracheae*,**,†, ‡	145 (24.2)	49 (32.7)	33 (22)	31 (20.7)	32 (21.3)
dorsal margin left atrium**	489 (81.5)	134 (89.3)	126 (84)	124 (82.7)	105 (70)
dorsal vena cava caudalis**,†, ‡	240 (40)	63 (42)	62 (41.3)	56 (37.3)	59 (39.3)
ventral vena cava caudalis*	116 (19.3)	29 (19.3)	30 (20)	23 (15.3)	34 (22.7)
apex cordis*,**,†	256 (42.7)	68 (45.3)	45 (30)	60 (40)	83 (55.3)
cranial heart side*	41 (6.8)	5 (3.3)	10 (6.6)	10 (6.6)	16 (10.7)

600 radiographic evaluations (50 per group and 200 per observer) were performed by three different veterinarians with different levels of experience (recently graduated veterinarian, experienced veterinarian, and specialized veterinarian); multiple selection possible. Observers rated the same radiographs. Staging according to the latest consensus statement of the American Colleague of Veterinary Internal Medicine (ACVIM).

*: part of VHS measurement.

**: part of RLAD measurement.

†: part of LA_{Width} measurement.

‡: part of VLAS measurement.

CG: Control group; LA_{Width}: Left Atrial Width; RLAD: Radiographic Left Atrial Dimension; VHS: Vertebral Heart Size; VLAS: Vertebral Atrial Size.

Additional Table f. Distribution of inverted well-identifiable radiographs and radiographs with measurement difficulties measured by the individual observers

Evaluation criteria	CG			Stage B1			Stage B2			Stage C		
	OB1	OB2	OB3	OB1	OB2	OB3	OB1	OB2	OB3	OB1	OB2	OB3
Well-identifiable radiographs												
VHS	12	13	27	22	18	27	15	18	31	12	10	27
RLAD	2	2	2	3	3	4	6	3	2	0	1	10
LA_{Width}	8	10	21	13	15	26	12	12	29	8	7	29
VLAS	20	14	25	25	19	27	29	18	30	30	18	32
anatomical landmarks												
carina tracheae*,**,†, ‡	15	18	16	5	14	14	8	13	10	7	14	11
dorsal margin of left atrium**	44	45	45	40	41	45	32	44	48	28	40	37
dorsal vena cava caudalis**,†, ‡	21	28	14	22	26	14	14	27	15	15	28	16
ventral vena cava caudalis*	12	12	5	11	12	7	7	7	9	8	15	11
apex cordis*,**,†	29	29	10	20	21	4	26	25	9	35	33	15
cranial heart side*	1	3	1	1	4	5	0	8	2	2	9	5

600 radiographic evaluations (50 per group and 200 per observer) were performed by three different veterinarians with different levels of experience (OB 1, OB 2, and OB 3); multiple selection possible. Observers rated the same radiographs. Staging according to the latest consensus statement of the American Colleague of Veterinary Internal Medicine (ACVIM).

*: part of VHS measurement.

**: part of RLAD measurement.

†: part of LA_{Width} measurement.

‡: part of VLAS measurement.

CG: Control group; LA_{Width}: Left Atrial Width; OB1: recently graduated veterinarian; OB2: experienced veterinarian; OB3: specialized veterinarian; RLAD: Radiographic Left Atrial Dimension; VHS: Vertebral Heart Size; VLAS: Vertebral Atrial Size.
