# Supplementary material for: Methods of Radiographic Measurements of Heart and Left Atrial Size in Dogs with and without Myxomatous Mitral Valve Disease: Intra- and Interobserver Agreement and Practicability of Different Methods
Source: Animals (Basel). 2022 Sep 22;12(19):2531. doi: 10.3390/ani12192531 (PMC9559670; doi:10.3390/ani12192531)
Supplement: Supplementary file 1 [file animals-12-02531-s001.zip › Supplementary File S4.pdf]

Additional Figure a. Measurement example of inverted VHS

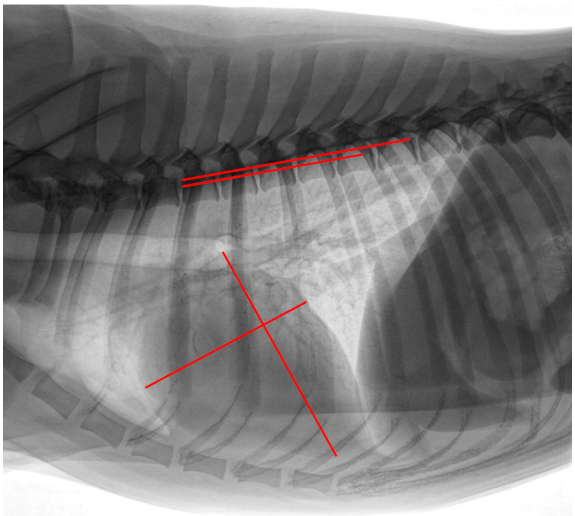

Description of data: Right lateral thoracic radiograph displaying the Vertebral Heart Size (VHS) measurement in a dog of the control group (same dog as in Figure 1). The long axis was drawn from the ventral margin of the carina tracheae to the most distal margin of the cardiac apex and perpendicular the short axis was drawn at the level of the ventral intersection of the caudal vena cava and the cardiac silhouette (red lines on cardiac silhouette). These lines were repositioned onto the vertebral column (red lines on vertebral column) beginning at the cranial margin of the fourth thoracic vertebrae. The VHS was 10.5 vertebral units.

Additional Figure b. Measurement example of inverted RLAD

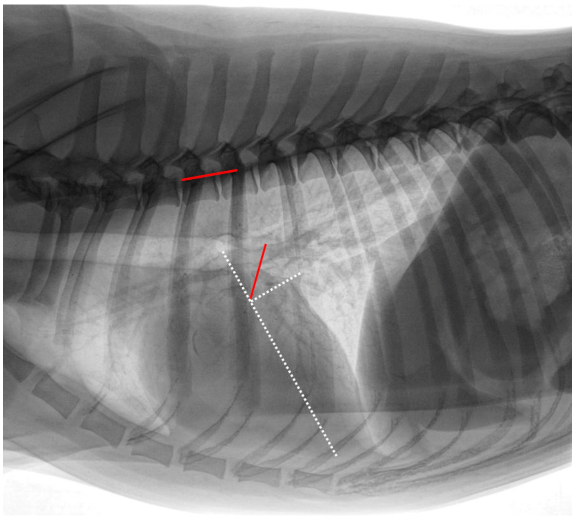

Radiographic Left Atrial Dimension (RLAD) measurement in the same inverted thoracic radiograph seen in Additional Figure a. The long axis (white dotted line) was applied as described for the VHS measurement (Figure 1). The short axis (white dotted line) was drawn from dorsal intersection of the caudal vena cava and the cardiac silhouette to the long axis. The bisecting RLAD line was drawn from the intersection point to the dorsal margin of the left atrium (red line on cardiac silhouette). This line was transposed onto the vertebral column (red line on vertebral) as described in Additional Figure a. The RLAD was 1.6 vertebral units.

Additional Figure c. Measurement example of inverted LA_{Width}

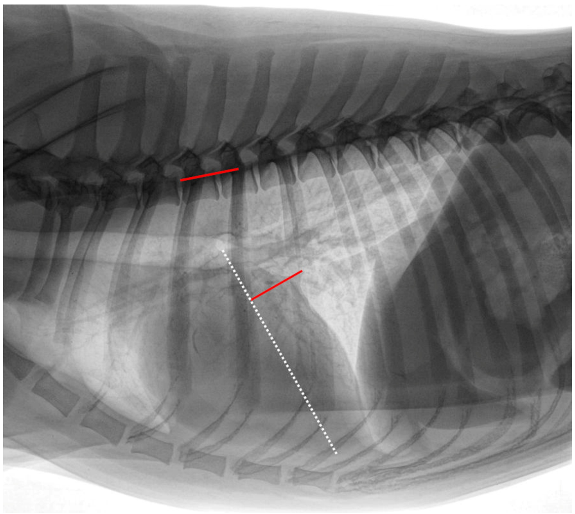

Left Atrial Width (LA_{Width}) measurement in the same inverted thoracic radiograph seen in Additional Figure a. The long axis was measured as described in Figure 1 (white dotted line). The short axis was drawn at a 90º angle to the long axis at the height of the dorsal intersection between the cardiac silhouette and the caudal vena cava (red line on cardiac silhouette). This line was repositioned onto the vertebral column (red line on vertebral) as described in Additional Figure a. The LA_{Width} was 1.6 vertebral units.

Additional Fig d. Measurement example of inverted VLAS

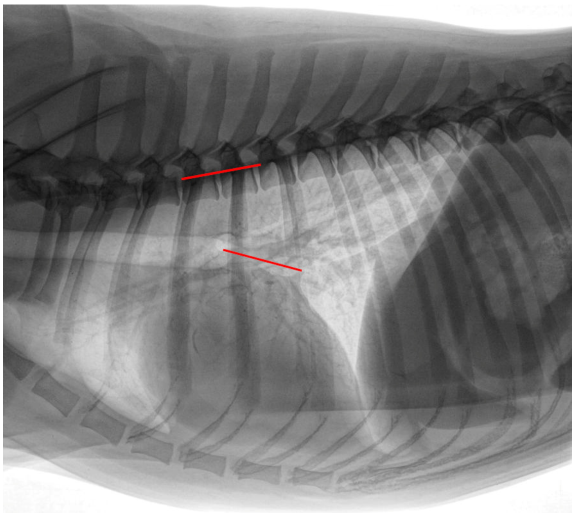

Vertebral Left Atrial Size (VLAS) measurement in the same inverted thoracic radiograph seen in Additional Figure a. A line was drawn from the ventral margin of the carina tracheae to the dorsal intersection between the cardiac silhouette and the caudal vena cava (red line on cardiac silhouette). This line was transposed onto the vertebral column (red line on vertebral) as described in Additional Figure a. The VLAS was 2.1 vertebral units.

Additional Figure e. Measurements of the radiographic measurements in conventional radiographs in the control group

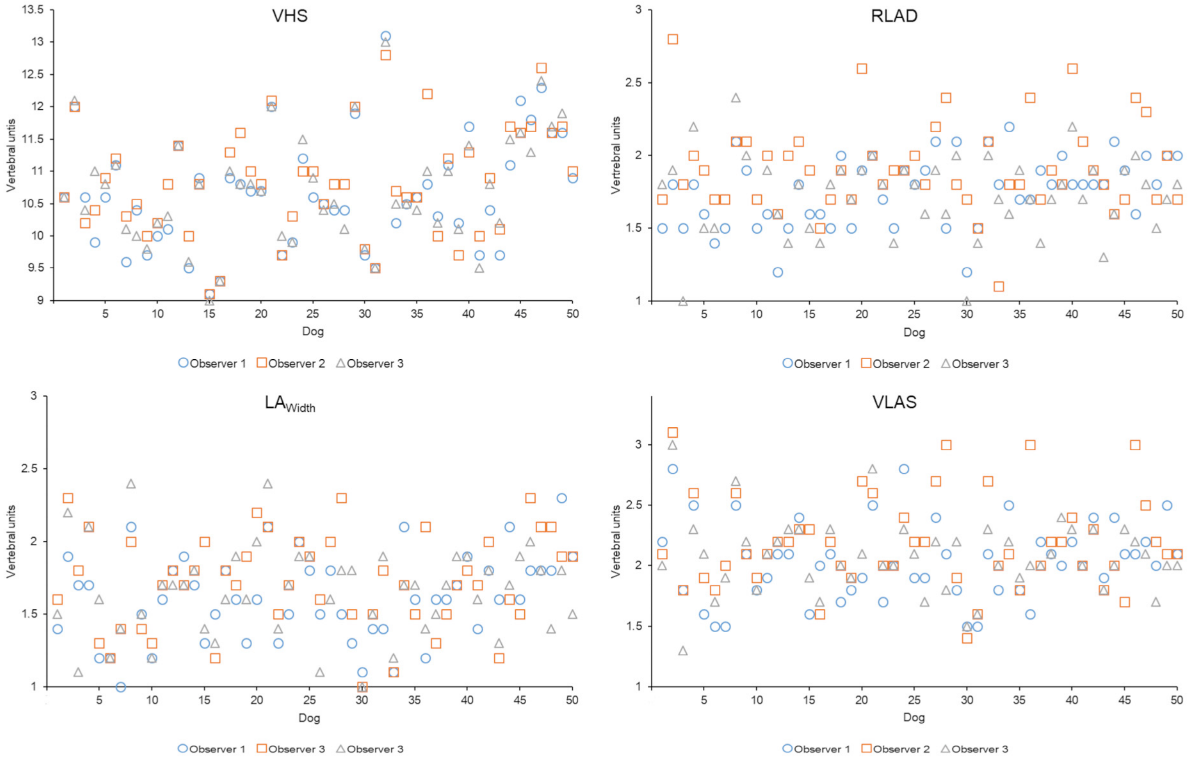

Fifty radiographs of the control group were examined by three different veterinarians with different levels of experience. The dogs (1 to 50) are displayed on the x-axis. The measurement of the various radiographic measurements (VHD, RLAD, LA_{Width}, and VLAS) in vertebral units can be seen on the y-axis. VHS measurement by the different observers are closer together per case compared to RLAD, LA_{Width}, and VLAS.

LA_{Width}: Left Atrial Width; Observer 1: recently graduated veterinarian; Observer 2: experienced veterinarian; Observer 3: specialized veterinarian; RLAD: Radiographic Left Atrial Dimension; VHS: Vertebral Heart Size; VLAS: Vertebral Atrial Size.

Additional Figure f. Measurements of the radiographic measurements in conventional radiographs in the stage B1

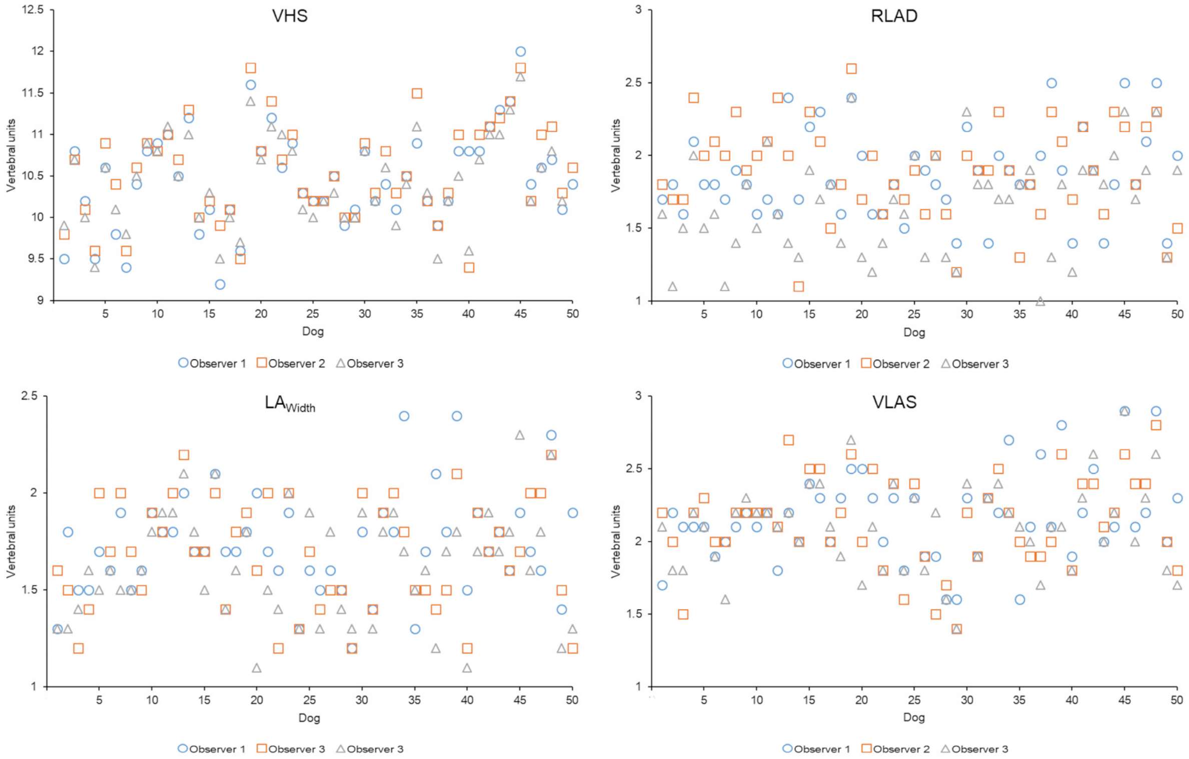

Fifty radiographs of the control group were examined by three different veterinarians with different levels of experience. The dogs (1 to 50) are displayed on the x-axis. The measurement of the various radiographic measurements (VHD, RLAD, LA_{Width}, and VLAS) in vertebral units can be seen on the y-axis. VHS measurement by the different observers are closer together per case compared to RLAD, LA_{Width}, and VLAS.

LA_{Width}: Left Atrial Width; Observer 1: recently graduated veterinarian; Observer 2: experienced veterinarian; Observer 3: specialized veterinarian; RLAD: Radiographic Left Atrial Dimension; VHS: Vertebral Heart Size; VLAS: Vertebral Atrial Size.

Additional Figure g. Measurements of the radiographic measurements in conventional radiographs in the stage B2

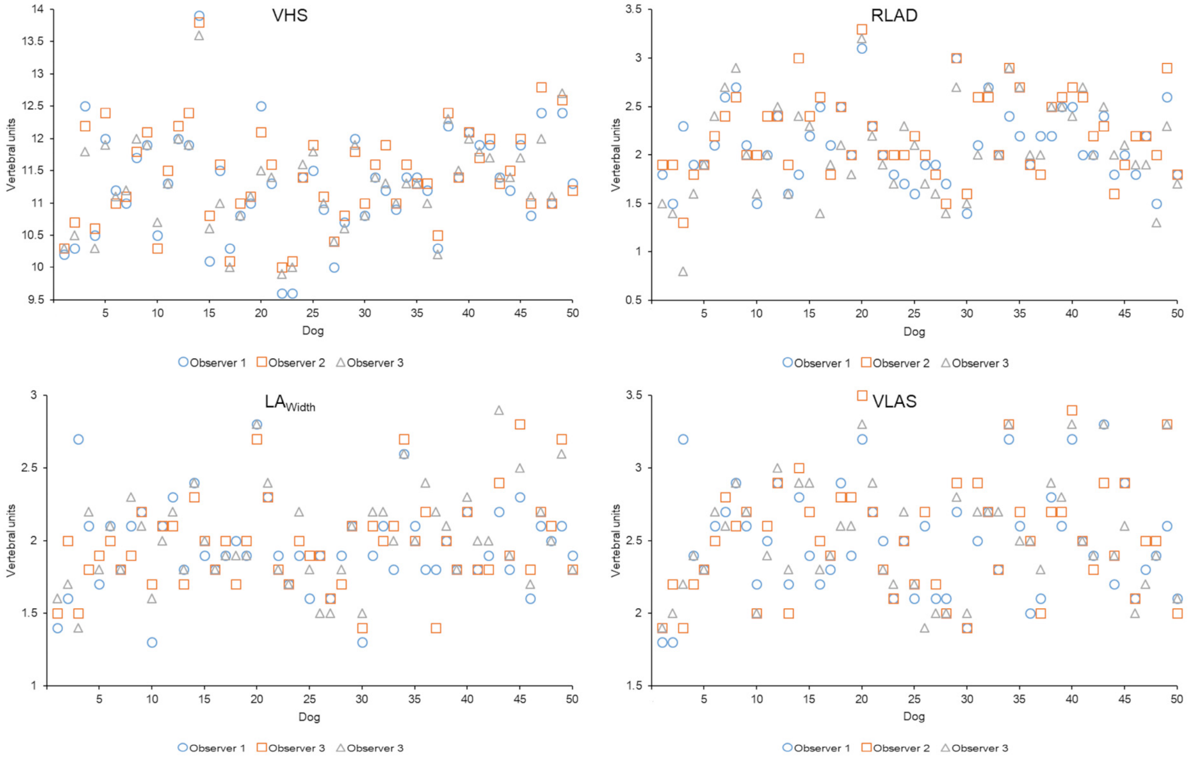

Fifty radiographs of the control group were examined by three different veterinarians with different levels of experience. The dogs (1 to 50) are displayed on the x-axis. The measurement of the various radiographic measurements (VHD, RLAD, LA_{Width}, and VLAS) in vertebral units can be seen on the y-axis. VHS measurement by the different observers are closer together per case compared to RLAD, LA_{Width}, and VLAS.

LA_{Width}: Left Atrial Width; Observer 1: recently graduated veterinarian; Observer 2: experienced veterinarian; Observer 3: specialized veterinarian; RLAD: Radiographic Left Atrial Dimension; VHS: Vertebral Heart Size; VLAS: Vertebral Atrial Size.

Additional Figure h. Measurements of the radiographic measurements in conventional radiographs in the stage B2

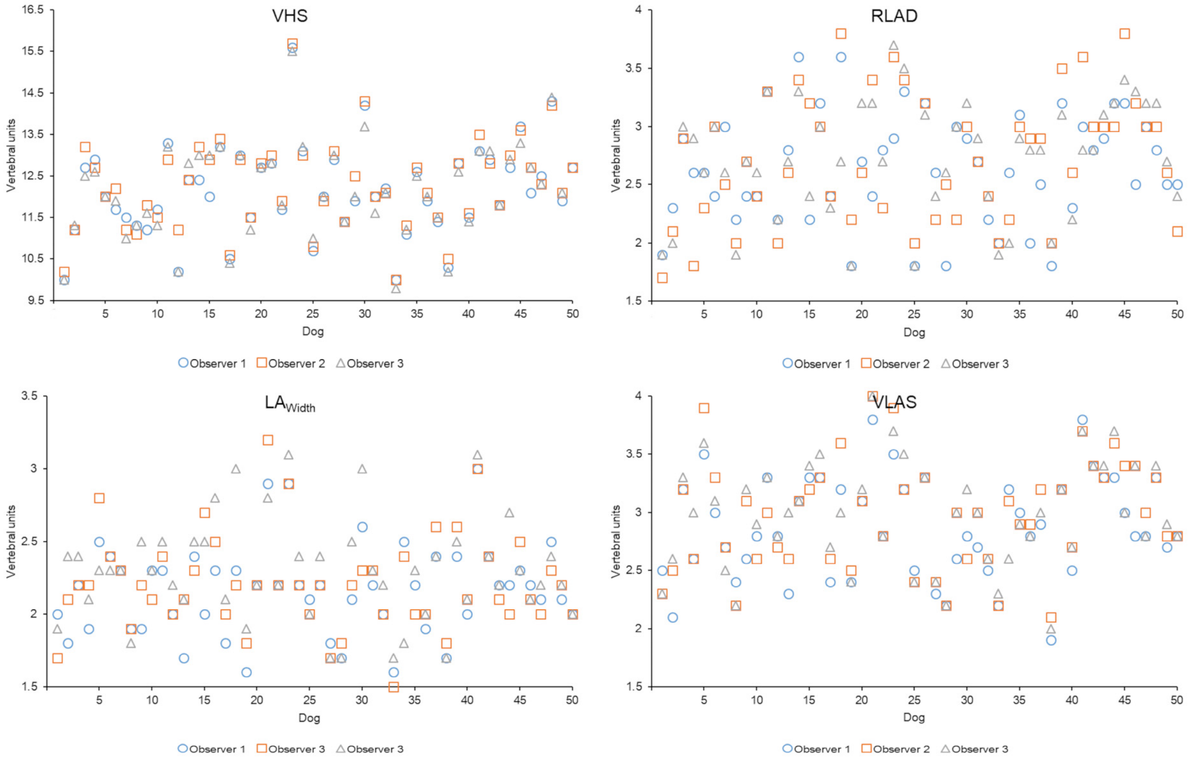

Fifty radiographs of the control group were examined by three different veterinarians with different levels of experience. The dogs (1 to 50) are displayed on the x-axis. The measurement of the various radiographic measurements (VHD, RLAD, LA$_{Width}$, and VLAS) in vertebral units can be seen on the y-axis. VHS measurement by the different observers are closer together per case compared to RLAD, LA$_{Width}$, and VLAS.

LA$_{Width}$: Left Atrial Width; Observer 1: recently graduated veterinarian; Observer 2: experienced veterinarian; Observer 3: specialized veterinarian; RLAD: Radiographic Left Atrial Dimension; VHS: Vertebral Heart Size; VLAS: Vertebral Atrial Size.
